# Ischemic colitis as a cause of severe hematochezia: A mini review

**DOI:** 10.46439/gastro.1.005

**Published:** 2022

**Authors:** Usah Khrucharoen, Dennis M. Jensen

**Affiliations:** 1 CURE Hemostasis Research Unit and the UCLA Digestive Diseases Research Core Center (UCLA: DDRCC), Los Angeles, CA, USA; 2 David Geffen School of Medicine at University of California, Los Angeles, CA, USA; 3 Vatche and Tamar Manoukian Division of Digestive Diseases and Department of Medicine, Ronald Reagan UCLA Medical Center, Los Angeles, CA, USA; 4 Division of Digestive Diseases and Department of Medicine, VA Greater Los Angeles Healthcare System, Los Angeles, CA, USA

**Keywords:** Ischemic colitis, Severe hematochezia, Colonoscopic hemostasis, Outcomes

## Abstract

Ischemic colitis (IC) is a common cause of severe lower gastrointestinal bleeding (LGIB) in the elderly. There are very few studies of patients with IC as a cause of severe LGIB in the literature. This article aims to review diagnosis, colonoscopic findings, medical treatment, and outcomes of patients with IC as a cause of severe hematochezia. The majority of IC patients with severe hematochezia can be successfully managed with medical treatment. Colonoscopic hemostasis with hemoclips is safe and feasible in treating major stigmata of recent hemorrhage in focal ischemic ulcers. Colon surgery is indicated in patients who fail medical treatment and/or have severe ongoing bleeding, clinical deterioration, or peritoneal signs. Overall, the morbidity rates in patients with IC range from 10% to 79%. Clinical outcomes in patients who need colon surgery for IC are worse than those treated with medical management. Patients who develop hematochezia from IC during hospitalization for other medical conditions have worse clinical outcomes than those with an outpatient start of bleeding. Further research is warranted for the prevention, early diagnosis, and treatment of patients with severe hematochezia from IC.

## Introduction

Ischemic colitis (IC) or colonic ischemia is a common diagnosis in elderly patients hospitalized for severe hematochezia [1,2]. IC has been increasingly diagnosed over the past 3 decades. The age-adjusted incidence rate increased from 6 to 23 cases/100,000 person-years from 1976–1980 to 2005–2009 [3]. Patients with IC and hematochezia can present with a variety of symptoms, depending on the location and severity of the ischemic injury. These include non-specific or severe abdominal pain, nausea, fecal urgency, severe hematochezia, and/or non-bloody diarrhea [4].

Several known predisposing factors associated with IC are old age, female gender, multiple comorbidities including hypertension (60% of IC patients), diabetes (24%), cardiovascular disease (24%), chronic kidney disease (15%), atrial fibrillation (14%), coagulation disorders, transient hypotension, and postsurgical abdominal aortic aneurysm repair or post cardiac surgery with extracorporeal circulation [3–11]. IC can also occur in younger patients with hypercoagulable states such as those associated with COVID-19 infection [12–15].

Several clinical patterns and classifications of IC have been described in literature [16–21]. Approximately 80–85% of patients have non-gangrenous colitis and the rest have gangrenous colitis [16–19]. IC can also be classified as reversible colopathy (3–26% of IC patients), transient IC (44%), chronic IC (18–25%), ischemic colonic stricture (10–15%), gangrenous colitis (0–19%), and universal fulminant pancolitis (1–2.5%) [19–21]. Another classification of the IC severity as mild, moderate, and severe has been clinically applied to patient management. This classification is based upon clinical signs and symptoms, risk factors for poor prognosis, colonoscopic findings, and laboratory values. Patients with moderate or severe IC are likely to require surgical intervention [22].

IC patients presenting to gastroenterologists (GI) with severe painless hematochezia are usually clinically distinguishable from those presenting to surgical services. The majority of GI patients with severe hematochezia are initially suspected of having transient colitis or reversible colopathy. They usually have painless hematochezia, lack peritoneal signs of transmural injury, and may have hemodynamic instability related to severe GI hemorrhage, but do not have sepsis, fever, or evidence of severe colon injury. Less than one-fourth of patients are initially suspected of IC from their clinical presentations [20]. Early colonoscopy is recommended for establishing the diagnosis and guiding management of IC [1,20,22]. In those with severe bleeding and stigmata of recent hemorrhage (SRH) on a focal ischemic ulcer, colonoscopic hemostasis can be safely performed [1,23].

In contrast, those patients presenting to surgical services usually have abdominal pain, abdominal rebound tenderness, septic shock, or absence of hematochezia and are suspected of having transmural ischemic injury or gangrenous colitis [1,24]. Clinical presentation and laboratory of IC can be difficult to evaluate. This is especially challenging in patients who are already hospitalized for other multiple severe medical conditions or in the intensive care unit and develop severe painless rectal bleeding.

Despite IC being a common cause of severe lower gastrointestinal bleeding (LGIB), there are very few reports in literature of these IC patients presenting with painless hematochezia [1,23,25]. Most patients with IC are reported in the surgical literature and present with severe IC, peritoneal signs, and not painless rectal bleeding. For patients with severe hematochezia from IC, our aims are to review diagnosis, colonoscopic findings, management, and clinical outcomes of medical and surgical treatments. We also compare risks and outcomes of those with outpatient vs. inpatient start of severe bleeding from IC.

## Colonoscopic Findings and Classifications of IC

**Figure 1:**
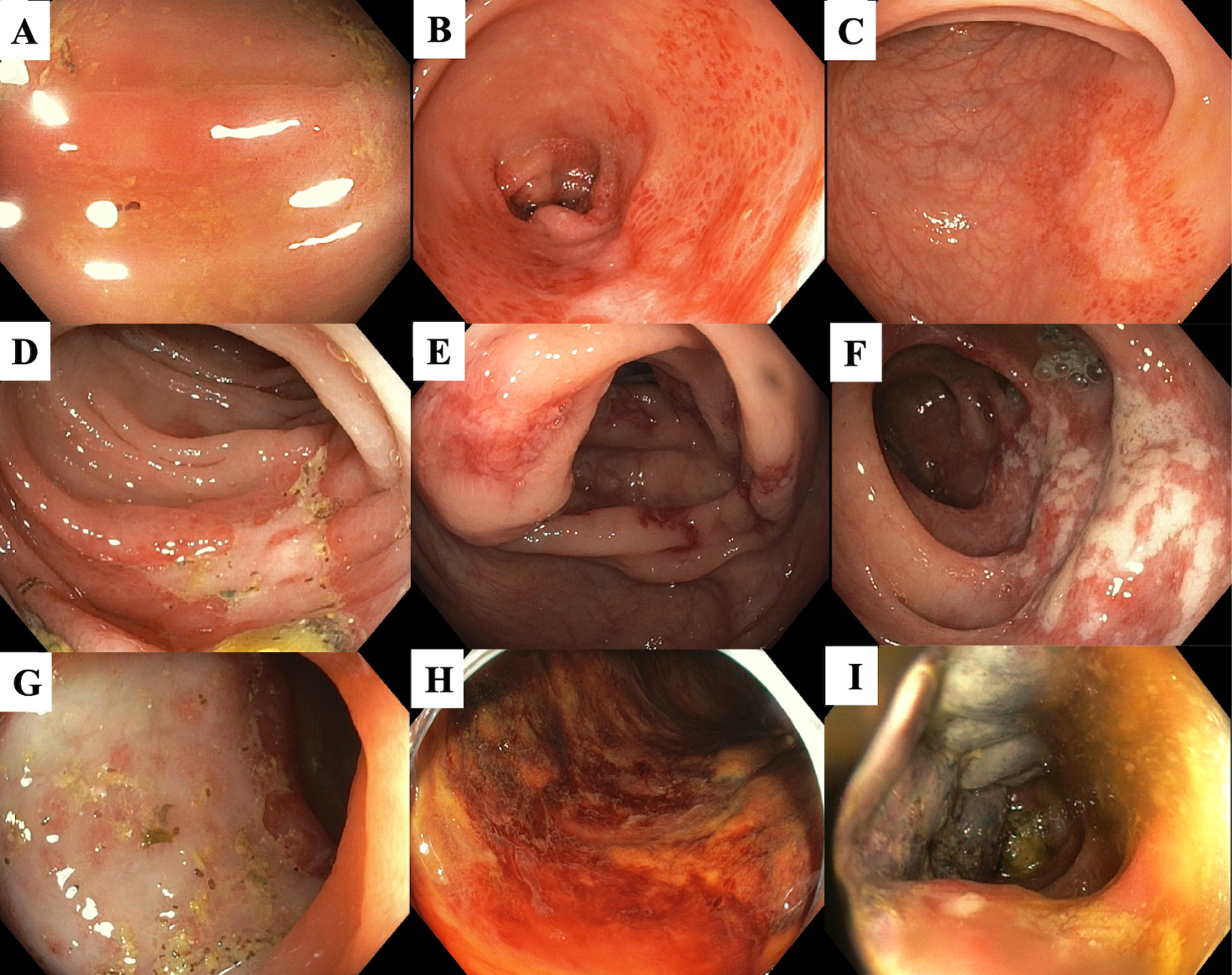
Recommended classification of ischemic colitis for risk stratification during colonoscopy. **(A)** Erythema and edematous mucosa; **(B)** Patchy erythema, petechial hemorrhage, and pale area; **(C)** Superficial longitudinal ulcer; **(D)** Irregularly shaped ulcer; **(E)** Scattered deep ulcerations; **(F)** Scattered ulcerations and mucosal edema along the longitudinal axis of the colon; **(G)** Near semi-circumferential ulcer; **(H)** Diffuse mucosal congestion and hemorrhage; and **(I)** Cyanotic mucosa and pseudopolyps [18,23]. [Colonoscopic images were from patients who previously enrolled in the CURE severe hematochezia studies with UCLA Institutional Review Boards approval].

Colonoscopic findings in patients with transient colitis and reversible colopathy are associated with mucosal or submucosal ischemic injury. These include segmental erythema and edematous mucosa, petechial hemorrhages, erosions, non-necrotic ulceration, and/or longitudinal ulcerations. In those with gangrenous colitis, colonoscopic findings include cyanotic mucosa and pseudopolyps [18]. Figure 1 shows colonoscopic findings of ischemic colitis, ranging from mild erythema to gangrenous mucosa.

Favier et al. first described an endoscopic classification of IC in 1976 by grading the colonoscopic severity into 3 stages [26]. Stage I includes patchy erythema, erosions, and small ulcerations with ischemia limited to the mucosa. Stage II includes larger and deeper non-necrotic ulcerations with ischemia limited to the submucosa. Stage III includes necrotic, gangrenous, and a possible perforated colon due to transmural injury [26]. The Favier classification is clinically useful in diagnosing and assessing the prognosis of IC and the need for surgery. Patients with stage III (IC in Figure 1) are associated with a high risk of requiring colon surgery and longer hospital stay [23,26–28].

In our recently published study of prospectively collected data for 97 histologically proven IC patients presenting with severe hematochezia, 72.2% of patients had Favier’s stage II on initial urgent colonoscopy. Others had stage I - 14.4% and stage III - 8.2%. Approximately 80% of IC patients treated medically had stage II, whereas 40% of patients undergoing colon surgery had Favier’s stage III [23].

In a retrospective study of 106 IC patients, Beppu et al. reported that those with longitudinal and circumferential ulcers (Stage III) had longer hospital stays than those with erythema and erosions [27]. In another retrospective study of 71 severe IC patients, Lorenza et al. reported that 24 patients had stage III. Of these, 68% required colectomy (p=0.028) [28].

## Medical Management for IC

According to the American College of Gastroenterology (ACG) Clinical Guideline for IC published in 2015, colonoscopy within 48 hours is strongly recommended in patients with a suspicion of IC. In most cases an IC diagnosis can be established from colonoscopic appearance. Biopsies can confirm the diagnosis. However, biopsies are not recommended in cases with colonoscopic gangrene because of a high risk of colon perforation. In patients with clinically severe IC, computerized tomography (CT) with intravenous and oral contrast or magnetic resonance imaging (MRI) is recommended to evaluate the severity and distribution of IC. In severe cases with possible transmural injury, limited colonoscopy with minimal air inflation is recommended. However, in patients with peritoneal signs or radiological evidence of transmural gangrene, pneumatosis, or perforation, colonoscopy is contraindicated [22].

In our experience, all patients with IC and severe hematochezia but without peritoneal signs can have safe and effective colonoscopy after purge. Almost 75% of our IC patients with severe hematochezia are successfully managed medically. Most of them have mucosal or submucosal, non-transmural colon injury. These patients usually have clinical improvement within 24–48 hours [23]. Initial medical management includes intravenous fluids, treatment of precipitating factors, packed red blood cell (PRBC) transfusion for severe bleeding (e.g. for hemoglobin < 8 g/dL), avoidance of vasoconstricting medications, supportive treatment, and bowel rest.

Although the majority of IC patients do not require antimicrobial therapy, broad-spectrum antimicrobial agents against anaerobes and gram-negative bacteria are recommended in those with moderate and severe IC due to an increased risk of bacterial translocation, inflammatory response to ischemia, and peritonitis [22]. Glucocorticoids have no role in treating IC patients, since these agents can potentially cause further ischemic damage, increase the risk of perforation, and reduce healing [22,29].

Surgery is reserved for those who fail medical treatment and/or have severe ongoing bleeding, progressive hemodynamic instability, peritoneal signs of colon perforation, or recurrent sepsis from IC [20,22,23,30,31]. For IC patients with severe painless bleeding from diffuse mucosal injury, very few have been treated with angiographic embolization [23]. Focal ulcers with major SRH can be treated with colonoscopic hemoclipping [1,23].

## Colonoscopic Treatment for IC

Although severe bleeding from focal ulceration with major stigmata of recent hemorrhage (SRH) in IC is uncommon, colonoscopic hemostasis with hemoclipping of major SRH in the bleeding ulcer is safe and effective [1,23]. Major SRH include active bleeding, non-bleeding visible vessel – NBVV, and adherent clot, similar to SRH identified in diverticular hemorrhage [1,23,32]. In IC focal ulcers with major SRH, hemoclipping is the preferred hemostasis method, because it does not cause significant tissue damage to the tissue [33]. Hemoclips can be directly deployed across the underlying artery, thereby reducing the risk of further bleeding [1,23,33]. Hemoclipping is also a safer technique for IC patients, especially in those with clotting abnormalities or those requiring anticoagulation. Thermal endoscopic hemostatic methods such as multipolar electrocoagulation (MPEC) or argon plasma coagulation (APC) may increase the risk of colon perforation by causing ulceration and if excessive thermal energy or excess gas insufflation is applied [33].

In our recently published study, 12.4% of 97 IC patients had focal ischemic ulcers with major SRH and were treated with colonoscopic hemostasis. Of these, 75% were treated with hemoclip and the rest were treated with a combination of hemoclip and MPEC, MPEC alone, and epinephrine injection alone. Among these patients, no adverse effects related to colonoscopic hemostasis occurred [23].

Hemostatic powder as a rescue treatment following failed hemoclipping and epinephrine injection on large oozing IC ulcers (25–50 mm in diameter) has been reported in a case series of 4 IC patients [34]. Hemostatic powder has a potential role of controlling diffuse bleeding in IC or from a large ulcer. However, it is temporary since the powder sloughs off in a few days and it also has an increased risk of GI perforation with IC due to its pressurized component. A case of gastric anterior wall perforation following the application of hemostatic powder has been reported [35]. There is still very limited evidence about the safety, efficacy, and indications of hemostatic powder for IC patients with LGIB [35].

## Outcomes in Patients with IC as a Cause of Severe Hematochezia

The morbidity rates in patients with IC are variably reported in the literature ranging from 10% to 79% [36]. However, most reports are from surgical type IC patients without severe hematochezia [36]. The mortality rates reported among patients who had surgery for IC are as high as 54%, depending on cohort (3.7– 54%) [30,36,37]. Higher disease-specific mortality rates are reported in patients with multiorgan failure. In patients with Favier’s stage III and multiorgan failure, the mortality rate was 65.8% compared to 16.6% without multiorgan failure. Similarly, in patients with Favier’s stage II, those with multiorgan failure had a higher mortality rate than those without multiorgan failure (53.3% vs. 0%). No mortality was reported among patients with Favier’s stage I [37].

Among IC patients with severe hematochezia, we recently reported that surgical patients had higher baseline comorbidity scores and more Favier’s stage III. Furthermore, patients who had colon surgery due to failed medical treatment (subgroup A) and those who were referred for surgery but not considered to be surgical candidates (subgroup B) had significantly worse clinical outcomes than those receiving medical treatment (subgroup C) [23]. Major clinical outcomes included longer hospital and intensive care unit (ICU) stays; more transfusions of PRBC’s [A. vs. B vs. C: median (IQR) – 5 (3–10) vs. 4.5 (3–6.5) vs. 1 (0–4) units]; higher severe complication rates (35.3% vs. 100%. vs 5.6%); and higher 30-day all-cause mortality rates (23.5% vs. 87.5% vs. 0) [23].

When comparing outcomes of IC patients with those bleeding from other colonic lesions, patients with IC had significantly worse 30-day outcomes than those with other colonic diagnoses. This was reported in our previously published study of 65 IC patients compared to 485 patients with other colonic diagnoses as a cause of severe hematochezia [1]. Other colonic diagnoses included non-ischemic colitis and hemorrhage from diverticulosis, arteriovenous malformations, colonic polyps, delayed post-polypectomy induced ulcers, and colon cancer. The 30-day clinical outcomes of IC vs. other colonic diagnoses included higher rates of 30-day rebleeding (27.7% vs. 12.6%), surgical intervention 13.9% vs. 5.6%), and longer ICU and hospital stays [1].

In a more recent study, we also compared outcomes in IC patients based upon start of severe bleeding - outpatient vs. inpatient (e.g. hospitalized for other medical or surgical conditions) [23]. Those with inpatient start of hematochezia (n=48) had higher baseline comorbidity scores, received significantly more PRBC transfusions, and had longer ICU and hospital stays than those with outpatient start of hematochezia (n=49). They also had a higher rate of colon surgery (27.1% vs. 8.2%), more severe complications, and higher 30-day all-cause mortality (18.8% vs. 4.1%) [23].

In another retrospective study, Nagata et al. reported outcomes of 57 IC patients with outpatient start of hematochezia compared to 313 patients with other colonic diagnoses as causes of hemorrhage [25]. They reported that IC patients with outpatient start of hematochezia had significantly better outcomes than those bleeding from other colonic diagnoses. Outcomes included fewer total PRBC transfusions (mean ± SD, 1.4 ± 8.8 vs. 2.6 ± 5.9 units) and shorter hospital stays (8.8 ± 3.6 vs. 13.1 ± 9.0). The rebleeding rates were also lower in IC patients (0 vs. 8%). However, the rates of surgical intervention were not significantly different (1.8% vs. 1.9%). During a mean follow-up of 22 months, IC patients also had a significantly lower rebleeding rate than those with other colonic diagnoses (5.3% vs. 19.4%) [25].

## Limitations and Gaps in Literature

There are very few reports of patients with IC as a cause of severe hematochezia in the literature [1,23,25]. Most studies cited in this review are small, retrospective studies. Although there are several strong recommendations in literature regarding the management of IC, most of these are based on surgical type patients without severe hematochezia [22]. For IC with severe hematochezia, more evidence-based data on primary prevention, early diagnosis, effective pharmacological treatment, and endoscopic hemostasis are lacking but needed to improve patient outcomes.

## Conclusions

IC is a common cause of severe hematochezia in elderly patients. Management of IC depends upon the severity of IC and patient’s clinical status. The majority of IC patients have non-transmural injury, transient colitis, and reversible colopathy. They can be successfully managed with medical treatment. Colonoscopic hemostasis using hemoclip is safe in selected patients with focal ischemic ulcers and major SRH. A small percentage of these patients with severe bleeding and advanced stage IC require surgery. However, some patients who are referred for surgery but are not surgical candidates due to multiple severe comorbidities. Clinical outcomes in those undergoing colon surgeries for IC and those who are not surgical candidates are significantly worse than in those treated medically. Also, patients with outpatient start of hematochezia have better clinical outcomes than those with inpatient start of bleeding.

## Abbreviations:

IC: Ischemic colitis
APC: Argon Plasma Coagulation
CT: Computerized Tomography
GI: Gastrointestinal
ICU: Intensive Care Unit
IQR: Interquartile Range
LGIB: Lower Gastrointestinal Bleeding
MPEC: Multipolar Electrocoagulation
MRI: Magnetic Resonance Imaging
PRBC: Packed Red Blood Cells
SD: Standard Deviation
SRH: Stigmata of Recent Hemorrhage
